# Strategies Used by Nurses to Maintain Person–Family Communication during the COVID-19 Pandemic: A Scoping Review

**DOI:** 10.3390/nursrep13030098

**Published:** 2023-08-21

**Authors:** Delfina Teixeira, Sandra Costa, Ana Branco, Ana Silva, Pablo Polo, Maria José Nogueira

**Affiliations:** 1 Departamento de Enfermagem, Escola Superior de Saúde de Santarém, Instituto Politecnico de Santarem, 2001-904 Santarém, Portugal; sandra.costa@essaude.ipsantarem.pt; 2 ULSAM—Unidade Local de Saúde do Alto Minho, Hospital de Santa Luzia, 4904-858 Viana do Castelo, Portugal; ana.branco@essantarem.pt; 3 Centro Hospitalar Póvoa de Varzim—Vila do Conde, Unidade da Póvoa de Varzim, 4490-421 Póvoa de Varzim, Portugal; ana.silva@essantarem.pt; 4 Hospital Montecelo, 36071 Pontevedra, Spain; pablo.polo@essaude.ipsantarem.pt; 5 Departamento de Enfermagem de Saúde Mental, Escola Superior de Enfermagem São João de Deus(ESESJD), Universidade de Évora, 7002-554 Évora, Portugal; maria.nogueira@uevora.pt

**Keywords:** family, communication, nurses, hospitals, COVID-19

## Abstract

**Background**: The COVID-19 pandemic made nurse–patient–family communication more difficult, reducing the understanding of the patient’s wishes and current care history. COVID-19 challenged healthcare teams to develop strategies to address these changes and provide more integrated care using the technology at their disposal. So, this study aims to map the strategies used by nurses to maintain communication between the person hospitalized with COVID-19 and the family to understand which communication technologies were most used to maintain communication between the person and the family. **Methods:** A Scoping Review, according to the recommendations of the Joanna Briggs Institute [JBI] with the Preferred Reporting Items for Scoping Review extension (PRISMA-ScR), research conducted between September 2022 and January 2023. The search was conducted in the databases: Latin American and Caribbean Literature in Health Sciences (LILACS); Cumulative Index of Nursing and Allied Health Literature (CINAHL); Scientific Electronic Library Online (SciELO); Medical Literature Analysis and Retrieval System Online (Medline), using the descriptors: family, communication, nurses, hospitals and COVID-19, and the Boolean operators “AND”. The inclusion criteria were: original articles, in Portuguese, English, and Spanish, published from 2020 onwards, with access to full and free text. **Results:** It was found that most of the communication was unstructured with the family. The technologies most used by nurses were the telephone with video calls from the patients themselves and even from health professionals to maintain communication between the patient and the family. **Conclusions:** Communication between patients and families became essential during the pandemic, as it became a vital lifeline of human connection that supported the mental health of patients and their families. This study was not registered.

## 1. Introduction

The pandemic caused by the coronavirus (COVID-19) has completely changed the concept of visiting health units, particularly in inpatient services with positive COVID-19 patients, introducing substantial communication barriers and abruptly hampering care centered on the patient and family [1]. Restricting family visits to institutions was deemed necessary due to concerns surrounding the transmission of the SARS-CoV-2 virus [1]. Thus, due to the need to maintain complete isolation, COVID-19 has largely changed the way nurses communicate with patients and families in COVID-19 care units. In addition, health professionals themselves were also isolated from their families and had to manage the consequences of this isolation, just like patients [2,3]. Also, during the COVID-19 pandemic reports, the academic literature and traditional media have highlighted the emotional and psychological negative impact on family members mainly due to visits restriction [2]. These restrictions made family members feel anguish and anxiety because they were kept away from sick family members at a time when they needed the most affection and support [4].

The absence of family members around the person infected with COVID-19 also harmed the patient’s recovery and psychological outcomes [4,5]. In addition, because the family members are often the best guardians of information about the patient’s continuity of care, wishes, and needs, the absence of family members hindered the transmission of important information about the patient’s past medical history, and current care history and communication, thus reducing the understanding of patient needs. As a result, this pandemic has challenged health systems, bringing multiple sectors together to provide more integrated care through technology. This had a vast impact on the healthcare system and the care-provided model [6]. The need for virtual visits and other digital health tools has increased dramatically to limit the transmission of the virus and alleviate an overburdened system [6]. Frontline workers were pressured to quickly adopt digital tools to safely and effectively deliver person-to-person care [7]. Despite the change in the adoption and use of digital tools to reduce the risk of virus transmission, there is limited data that explore the strategies used by nurses to maintain person–family communication during the pandemic, communication represents the basis of the nurse-family relationship.

Person–family communication constitutes the main mechanism for sharing experiences, feelings, and perceptions, as well as clarification, interaction, and knowledge, on the part of the nurse, to the family of the person in a hospital situation [8]. To manage communication, nursing teams resort to strategies to achieve interaction between the hospitalized person and the family; however, resorting to these strategies is a challenge for professionals, as it requires high levels of literacy in digital technologies to that this communication is established effectively [9]. Thus, a complete understanding of which strategies were implemented by nurses to maintain the nurse–person–family relationship and which communication technologies were most used to maintain it is necessary, realizing that there were several challenges they faced in adopting virtual communication during the COVID-19 pandemic [8,10]. The absence of studies about the techniques used by nurses to establish communication between the person and the family during the period of the COVID-19 pandemic, along with the need to empower nurses for this new reality, justifies the pertinence of investigating this topic. In this sense, we start with the question: *What are the strategies used by nurses to maintain person–family communication for those hospitalized in terms of COVID-19 services?* To answer this question, we defined the following objectives: to map strategies used by nurses to maintain communication between the person hospitalized with COVID-19 and the family and understand which communication technologies are most used to maintain communication between the person and the family.

## 2. Materials and Methods

The Scoping Review follows the guidelines of the Joanna Briggs Institute (JBI) [11] and the PRISMA-ScR extension through the five methodological stages [11]. The article selection protocol began with the 1st stage: identification of a problem and a research question using the PCC acronym [P (Population = nurses); C (Concept = strategies used by nurses to maintain person–family communication] and C (Context = hospitalized in COVID-19 services)]. In the 2nd stage, relevant studies were mapped. The research took place between September 2022 and January 2023, on the databases: Latin American and Caribbean Literature in Health Sciences (LILACS); Cumulative Index of Nursing and Allied Health Literature (CINAHL); Scientific Electronic Library Online (SciELO); Medical Literature Analysis and Retrieval System Online (Medline). DeCS descriptors [Communication; COVID-19; Family Relations; Nurses role, Hospitals] and MeSH descriptors [Communication; COVID-19; Family; Nurses; and Hospital] were used. The studies were selected using the Boolean operator “AND” and “OR” with the following formula using DeCS descriptors: Family Relations AND communication AND nurse’s role AND hospitals AND COVID-19”; and using MeSH descriptors: PubMed(&quot;Communication*&quot;) AND (&quot;Nurses*&quot;) AND (&quot;Hospitals*&quot;) AND (&quot;COVID-19*&quot;) = 161; (&quot;Family*&quot;) AND (&quot;Communication*&quot;) AND (&quot;Nurses*&quot;) AND (&quot;Hospitals*&quot;) AND (&quot;COVID-19*&quot;) = 48. Search: (Family*&quot;) AND (&quot;Communication*&quot;) OR (&quot;Nurses*&quot;) AND (&quot;Hospitals*&quot;) AND (&quot;COVID-19*&quot;) = 0. Search: (Family*&quot;) AND (&quot;Communication*&quot;) OR (&quot;Nurses*&quot;) AND (&quot;Hospitals*&quot;) AND (&quot;COVID-19*&quot;) = 0. Search: (Family*&quot;) OR (&quot;Communication*&quot;) OR (&quot;Nurses*&quot;) OR (&quot;Hospitals*&quot;) AND (&quot;COVID-19*&quot;) = 0. Search: &quot;communication*&quot; [All Fields] AND &quot;nurses*&quot; [All Fields] AND &quot;hospitals*&quot; [All Fields] AND &quot;COVID 19*&quot; [All Fields] = 161 results. Search: (&quot;Family*&quot;) AND (&quot;Communication*&quot;) AND (&quot;Nurses*&quot;) AND (&quot;Hospitals*&quot;) AND (&quot;COVID-19*&quot;) &quot;family*&quot; [All Fields] AND &quot;communication*&quot; [All Fields] AND &quot;nurses*&quot; [All Fields] AND &quot;hospitals*&quot; [All Fields] AND &quot;COVID 19*&quot; [All Fields] = 48. Subsequently, a search was carried out on Google Scholar with natural terms *thesaurus* synonyms (strategies used by nurses to maintain communication between people hospitalized in COVID-19 services and the family).

In the 3rd stage, articles were selected according to the previously defined inclusion criteria: original articles, in Portuguese, English, and Spanish, published from 2020 onwards, with access to a full and free text, which answered the guiding question. The references obtained were managed through Mendeley software, and duplicates were removed. The selection of the articles was carried out by three reviewers, through the reading of the title and/or abstract excluding the records that did not cover the inclusion criteria. In the 4th stage, the analysis and compilation of the information contained in the eligible articles were analyzed, and duplicated articles were excluded, whose title was not suggestive of the intended theme, as well as articles that did not allow their full reading.

**Figure 1:**
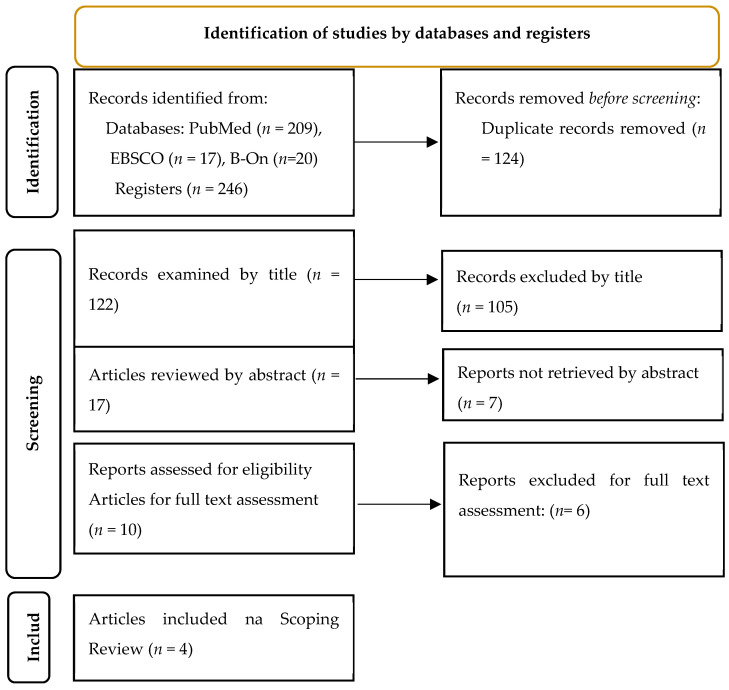
PRISMA 2020 flow diagram [11].

The selection of studies strictly followed the recommendations of the PRISMA-ScR, so no former quality assessment was made in the included studies. Our data extraction and analysis focused on a descriptive approach. After selection by title and abstract, the articles were carefully chosen for full-text reading, analyzed by the same reviewers, related to trends, gaps, and patterns in the literature, and afterward summarized and recorded the data collected in standardized evidence sheets organized according to the following items: author(s) year/country, title, design, aim, and results. The completion of these sheets enabled the identification of relevant articles for this Scoping Review, since when compiling the information, articles were also identified that only the full-text reading allowed to verify the presence of reliability conditions related to the exclusion criteria. Finally, in the 5th stage, data of interest were extracted in a summarized form and the results were reported. The search strategy and the different stages of selection, eligibility, and inclusion of articles are shown in Figure 1.

The flowchart of identification, selection, and compilation of articles is presented in Figure 1.

## 3. Results

The characterization of the articles included in this review is summarized based on the author(s), year, country of origin, title, base where it was located, study design, objective, and main results, and is presented in Table 1.

Table 2 summarizes the main communication strategies and tools used by nurses to maintain person–family communication during COVID-19. The articles were numbered from 1 to 4 for better identification in the text.

## 4. Discussion

The emergency caused by the COVID-19 pandemic has changed the way nurses communicate with the patient and families in all COVID-19 care units, due to the need to maintain complete isolation. Table 2 summarizes the strategies used by nurses to maintain communication between hospitalized patients and their families during the COVID-19 outbreak. The main results highlight strategies such as communication groups; outlining and sharing consensus statements to enable the health care team to provide an optimal level of communication with patients’ families in circumstances of total isolation; phone calls in an attempt to respond to the families’ expectations; start the family visit to the patient in a virtual format; organizing telehealth visits played an important role in facilitating communication between patients and families; implementation of a reservation system to schedule calls and meetings with family members, health care providers, using interpreters when necessary; creation of family liaison nurses to facilitate communication between the hospitalized patient and the family; and phone calls with unstructured communication with the family, often depending on individual actions by the family or the nurse responsible for the patient [1,2,3,4].

In a pandemic emergency, the accessibility of information must be a priority, and in this context, nurse–patient–family communication must flow reciprocally, because good communication is a guarantee that essential information (for care delivery) has been received and understood by healthcare professionals. Similarly, relevant, pertinent, and clear information about patient conditions must be delivered to the family member, which provides security and comfort. Thus, in the COVID-19 context, everyone involved in the care process must be the clearest possible in communicating their information to avoid ambiguities and misunderstandings, especially because the therapeutic value of communication is severely limited [7,13,14].

Evidence increasingly supports the impact of family presence on improving patient care experiences and outcomes and on the mental and physical well-being of patients, families, and healthcare professionals [15,16,17,18]. Results highlighted that communication with the patient and family of the hospitalized patient is extremely important, as it is crucial to reduce anxiety and anguish (both in families and hospitalized patients) [14,15]. These findings are in line with current research that supports the central role of assertive communication in healthcare and clinical decision making [14,15,16,17]. In addition, our findings reveal that virtual visits offered an alternative way to [1] restore family unity, [2] facilitate family involvement, and [3] allow for the expression of the family’s feelings.

Virtual visits made it possible for multiple family members to simultaneously communicate and interact with the hospitalized relative and restored a sense of family unity [12,16,17]. Also, the family involvement level in end-of-life presence and the communication guidance activities, highlighted how the virtual visit could contribute to effective family-centered care [7,16]. Additionally, virtual visits were emotionally challenging for many family members, but also cathartic in helping them understand their own emotions and experiences, visualizing their hospitalized family members [7,16].

Several studies report that at the beginning of the COVID-19 outbreak, families felt that they were losing vital information, so communication with the health team was very important for them. They also felt some anguish since this information was not standardized or structured, depending on the different nurses in charge [5,7,16,17,18,19]. Some family members took the opportunity to be updated on their patient clinical status during phone call contact with healthcare professionals and some studies described these experiences as “disappointing”, “excellent” or “impersonal”, and express that communication with nurses was generally more consistent than communication with physicians [5,12,19].

Study results show that the technologies most used by nurses to maintain communication between the person and the family were phone calls with video, made by the patients themselves, or made by healthcare professionals. Some studies describe ambiguous feelings reported by family members about phone or video calls. Families often describe mixed feelings, both as “very satisfied” as well as “disturbing” and “traumatic” [5,7,13]. For health professionals, it was also a great challenge, adding this procedure to the many stressful routines in the ward units’ daily COVID-19 care. Many nurses had to deal with families’ constant phone calls to ask for updates on the health status of patients, and in the vast majority of hospitals, there was no management group to make daily updates to families [5,7,9,13].

Results show that several family members called the units several times a day to speak with the nurse in charge of their relative acre; however, others waited for the nurse to call them back to update the patient’s condition [5,13,16]. In the second example, the anxiety and fear reported by these families were particularly high, with many family members reporting that waiting for this phone call was like “torture” [5].

Additionally, it was found that using telehealth was another strategy used to maintain communication between the person and the family, especially when the family was conscious [4,5,18]. There are reports in some family studies that seeing family members via telehealth was good; however, for other families, it was a less positive disturbing experience [4,5,13]. The platforms most used to carry out communication were TouchAway, developed by the LifeLines team, followed by Skype and FaceTime. Despite these platforms not being the ideal way to maintain essential communication between the patient and the family, during the COVID-19 pandemic, they were the vital lifeline of human connection that supported the mental health of patients and their families [17]. In this context, some studies reported that these platforms and new technologies developed a key role promoted meaningful connections with their families and allowing them to feel present during the person’s hospitalization [4,9,17].

In this sense, some participants mentioned directly observing the negative impact of social isolation on the patient’s well-being and recovery during the period of visitation restrictions, saying that they could see that their loved one “did not want to live like this”. Ultimately, patients and families perceived that new technologies were a patient and family-centered innovation that can facilitate regular connection between them, not just during the COVID-19 pandemic situation, but at any time the patient needs care and comfort from and a connection with their family [9,16].

## 5. Conclusions

The analysis of the studies included in this review showed that the main strategies used by nurses to maintain communication between patients and families during the COVID-19 outbreak were phone calls. Performing a virtual visit played an important role in facilitating emotional support; however, these strategies wore unstructured, depending on the individual actions of the family or the nurse in charge of the patient. The technologies most used by nurses were the telephone–video calls, made by the patients themselves or by health professionals.

Maintaining communication between the patient and family played a central role during the COVID-19 pandemic, as it become a vital lifeline of human connection and promoting the mental health of the patient and family. This study also highlights the potential of new technologies as resources for nursing care, in a critical period for humanity, namely lessening the negative effects of isolation and/or limitation of significant relatives with patient contact. Finally, this study highlighted that nursing teams working during the COVID-19 context were able to find pioneering strategies to maintain and strengthen nurse–patient–family communication, considering it an essential guarantee in the provision of care.

## Figures and Tables

**Table 1:** Characterization of the articles included in this review.

Nº	Author(s) Year/Country	Title	Design	Aim	Results
1	Avellaneda-Martínez, et al. 2020, Espain [12]	Management of communication between inpatients isolated due to COVID-19 and their families	Qualitative with action research methodology	Describe the process designed to facilitate communication between patients hospitalized and isolated due to COVID-19 with their families, within the hospital’s quality and humanization plan.	The nursing team, in collaboration with the nursing management, proposed the creation of a group of managers to carry out communication between the hospitalized person and the family. Draft and share consensus statements to enable the healthcare team to provide, via telephone or video calls, an optimal level of communication with patients’ families in circumstances of total isolation. Communication aims to inform the patient’s clinical status and respond to the expectations of families.
2	Rose, L., Yu, L., Casey, J., Cook, A., Metaxa, V., Pattison, N., Rafferty, A.M., Ramsay, P., Saha, S., Xyrichis, A., Meyer, J., UK [9]	Communication and Virtual Visiting for Families of Patients in Intensive Care during the COVID-19 Pandemic: A UK National Survey	Multicêntric Study	Understand how communication between families, patients, and the ICU team was possible during the pandemic. Understand the strategies used to facilitate virtual visits and the benefits and barriers associated with this methodology.	Videoconferencing technology, and the challenges associated with family members’ ability to use videoconferencing technology or access a device. The most used platforms were the personalized version of the virtual visit platform, TouchAway, developed by the LifeLines team (43, 41%), followed by Skype (27, 25%), and FaceTime (24, 23%). All hospitals started the family visit to the patient in a virtual format. Commonly reported benefits of virtual visits were a reduction of patients’ psychological distress (78%), improved staff morale (68%), and reorientation of patients with delirium (47%).
3	Chen, R.T., Truong, M., Watterson, J.R., Burrell, A., and Wong, P. 2023, Australia [13]	The impact of the intensive care unit family liaison nurse role on communication during the COVID-19 pandemic: A qualitative descriptive study of healthcare professionals	A qualitative descriptive study	This study aimed to explore the impact of the role of the ICU Family Liaison Nurse on communicating with patients and their families during the COVID-19 pandemic, from the perspective of ICU healthcare professionals.	Participants felt that telehealth was a great help and played an important role in facilitating communication between patients and their families. It was relatively easy and less time-consuming to organize a telehealth meeting than an in-person meeting. Telehealth allowed families to see the faces of their loved ones and what is happening in the ICU, which was especially important during the closure. They again reported that communication was more regular and organized during the pandemic with the use of telehealth. A reservation system was implemented to schedule calls and meetings with family members, healthcare providers, and interpreters. Family liaison nurses were created to facilitate communication between the hospitalized patient and the family.
4	Maaskant, I.P. Jongerde, J. Bik, M. Joosten, S. Musters, M.N. Storm-Versloot, J. Wielenga, A.M. Eskes, on behalf of the FAM-Corona Group 2021, the Netherlands [7]	Strict isolation requires a different approach to the family of hospitalized patients with COVID-19: A rapid qualitative study	Qualitative study, using a retrospective review of patient records and semi-structured focus group interviews.	Investigate how family involvement had occurred, and explore nurses’ experiences with family involvement during the COVID-19 outbreak; formulate recommendations for family involvement.	The contact between the family and the health professionals was done mainly through video or telephone calls. An important precondition for contact between patients, their families, and/or healthcare professionals was the availability of telephones and the ability to use them. Most units had unstructured communication with the family, often depending on individual family or bedside nurse actions. In the focus groups, nurses explained that contact with families was much less than before the COVID-19 outbreak. the restricted visitation policy resulted in the absence of family, and (informal) communication stopped.

**Table 2:** Communication strategies and tools used by nurses to maintain person–family communication during COVID-19.

**Communication** **Strategies**	Creation of a group of managers to carry out the communication: 1; 4.
	Draft and share consensus statements to enable the healthcare team to provide an optimal level of communication with patients’ families in circumstances of total isolation: 1.
	Phone calls to try to meet the expectations of families: 1; 2; 3; 4.
	Visita virtual—Os hospitais iniciaram a visita da família ao doente em formato virtual: 1; 2; 3; 4.
	Organizar visitas de telehealth foi uma grande ajuda e desempenhou um papel importante na facilitação da comunicação entre doentes e familiares: 3.
	Implementing a reservation system to schedule calls and meetings with family members, healthcare providers, and interpreters: 3.
	Family liaison nurses were created to facilitate communication between the hospitalized patient and the family: 3.
	Phone calls with unstructured communication with the family, often depending on individual actions by the family or the nurse responsible for the patient: 1; 2; 3; 4.
**Communication** **Tools**	O telefone ou videochamadas, foram as tecnologias para manter a comunicação com os familiares dos doentes em circunstâncias de isolamento total 1; 4.
	Videoconferencing technology or accessing a device. The most used platforms for the virtual tour were TouchAway, developed by the LifeLines team, followed by Skype and FaceTime: 2.
	Telehealth was a great help and played an important role in facilitating communication between patients and their families: 3.

## Data Availability

Data can be made available upon request.

## Public Involvement Statement

No public involvement in any aspect of this research.

## Guidelines and Standards Statement

This manuscript was drafted against the *PRISMA2020 statement* for *reporting systematic reviews*.
